# Moderation Effects of Ethnic-Racial Identity on Disordered Eating and Ethnicity Among Asian and Caucasian Americans

**DOI:** 10.3389/fpsyg.2021.594391

**Published:** 2021-04-16

**Authors:** Katrina T. Obleada, Brooke L. Bennett

**Affiliations:** ^1^Department of Psychology, University of Hawai‘i at Manoa, Honolulu, HI, United States; ^2^Department of Psychology, Yale University School of Medicine, New Haven, CT, United States

**Keywords:** centrality, weight, shape, ethnic-racial identity exploration, disordered eating and exercise behaviors, in-group affect

## Abstract

**Background:** The current study was designed to examine whether ethnic-racial identity (ERI) moderated the relationship between disordered eating and primary ethnic identification.

**Methods:** Three hundred and ninety-eight undergraduate women (*M*_*age*_ = 19.95, *SD* = 3.09) were recruited from a large university in Hawai‘i. Participants completed the Eating Disorder Examination Questionnaire (EDE-Q), the ERI measure, and reported their primary ethnicity as an index of ethnicity.

**Results:** There was a significant correlation between eating concerns and centrality, *r*_(357)_ = 0.127, *p* < 0.05. Moderation analyses indicated that only ERI centrality moderated the predictive effect of ethnicity on the importance of eating concerns, *b* = 0.05, *t*_(347)_ = 2.37, *p* = *0.0*18.

**Conclusions:** The results suggest that the relationship between self-reported primary ethnicity and EDEQ scores is greater when ethnicity is more central to the individual's identity or when the in-group affect is important to an individual. Findings underscore the need for further research on the underlying mechanisms that account for the differing ways that ERI may affect eating concerns.

## Introduction

Eating disorders are serious illnesses, characterized by preoccupation with food and overvaluation of weight and shape, that cause severe psychological distress, role impairment, physiological problems, and interpersonal difficulties (Fairburn, 2008; Smink et al., 2012). Anorexia nervosa (AN), bulimia nervosa (BN), and binge-eating disorder (BED) are the three principal eating disorder diagnoses; however, it is also common for individuals to suffer from disordered eating (e.g., restriction, binge-eating, self-induced vomiting, compensatory exercise, and laxative and diuretics misuse) that does not meet the full criteria for these categories (Other Specified Feeding and Eating Disorder: OSFED). In the United States, lifetime prevalence rates for eating disorders are substantial, ranging from 0.6 to 4.5% and affecting mostly young women; lifetime prevalence rates for OSFED are estimated at 4.78% in adolescents and 4.64% in adults (Hudson et al., 2007; Le Grange et al., 2012). Research has shown that these conditions are also a global health issue, although they appear to be more prevalent in Western countries (Hoek, 2016).

Of particular interest is the dearth of research on their development and maintenance within Asian American populations. According to Nicdao et al. (2007), eating disorders affect Asian Americans at lower prevalence rates than the United States population as a whole. Asian Americans are less likely to seek treatment for psychological problems in general, however, suggesting that prevalence rates may be underestimates (Smart et al., 2011). Eating disorder pathology may also take somewhat different forms within samples of Asian American women (Thomas et al., 2016). Wardle et al. (2006) found that Asian women held stricter standards about achieving the values of thinness compared to other ethnicities. Despite having lower average body mass relative to Caucasian American women, Asian American women report higher levels of body dissatisfaction (Mitz and Kashubeck, 1999; Koff et al., 2001; Yates et al., 2004). Some investigators note that it can be difficult to diagnose using Western criteria within Asian national samples with eating disorders because Asian women tended not to endorse fears of fat or weight gain (Lee et al., 2010; Nakai et al., 2014a).

### Risk Factors for Eating Disorders

Research on risk factors suggests a complex interaction of biological (Striegel-Moore and Bulik, 2007) and genetic vulnerabilities (Bulik et al., 2016), temperamental variables (Lilenfeld, 2011; Wagner and Vitousek, 2019), family dynamics (Striegel-Moore et al., 1986), and general sociocultural pressures (Striegel-Moore et al., 1986; Wertheim and Paxton, 2011). Though these psychosocial risk factors have been studied mostly in Western and White populations, research has suggested similar correlates in other national, racial, and ethnic groups (Keel and Forney, 2013; Pike et al., 2020). According to Striegel-Moore and Bulik (2007), “the single best predictor for developing an eating disorder is being female” (p. 182). Adolescence and young adulthood appear to be especially vulnerable times, possibly due to pubertal changes and fat accumulation; this is peak age of onset for clinical eating disorders and is the developmental period, which highest scores are obtained in population-wide studies (Crisp and Kalucy, 1974; Rosenbaum, 1979; Attie and Brooks-Gunn, 1989; Hsu, 1990). Individuals at these life stages experience particular challenges (e.g., puberty-related physical changes amid cultural pressures to maintain a prepubertal body type, pursuit of personal and physical autonomy from parents, identity and personality development) that may make them vulnerable to eating problems (Attie and Brooks-Gunn, 1989).

### Other Unique Risk Considerations for Asians

According to Kawamura (2002), risk factors for body image distortion and disordered eating among Japanese women include adherence to a strict and distinct social hierarchy, emotional restraint, need for social approval, and desire to avoid bringing shame on the family. High parental expectations have been identified as a common risk factor, which subsumes desire to gain parental approval; family recognition through achievement in academics, career, and/or appearance; and management of family criticism about weight, eating, and appearance (Peng and Wright, 1994; Pike and Borovoy, 2004; Ting and Hwang, 2007; Isono et al., 2009; Chang et al., 2014; Smart and Tsong, 2014; Tsong and Smart, 2015). In addition, Asian American women may be subjected to sexist and racial stereotypes, such as assumptions that they should be petite or traditionally feminine, or the expectation that they should meet Western standards for physical appearance (e.g., facial features, skin color, height) that are difficult or impossible for Asian women to fulfill (Hall, 1995). Due to the additional pressure from cultural values, Asian American women must navigate traditional Asian values and mainstream American culture, possibly making them more vulnerable to body dissatisfaction and disordered eating (Wonderlich et al., 2007; Tsong and Smart, 2015).

### Ethnic Identity and Disordered Eating

Of particular interest is the relationship between ethnic identity and disordered eating. Previous research has produced mixed findings. Some results indicate a negative association (Henrickson et al., 2010; Stein et al., 2010) or no association between ethnic identity and eating pathology (Yoshimura, 1995; Stark-Wroblewski et al., 2005). Other studies suggest that ethnic identity may serve as a protective factor against thin-ideal internalization and eating pathology (Rhea and Thatcher, 2013; Rakhkovskaya and Warren, 2014).

Ting and Hwang (2007) proposed that intergenerational cultural strain can occur when acculturated daughters misunderstand their parents' expressions of love and care due to culturally normative parenting, such as criticism and shame regarding eating, weight, and appearance. Consistent with this analysis, Lau et al. (2006) found that Asian Americans with higher levels of body dissatisfaction endorsed a stronger identification (i.e., centrality) with traditional Asian values. It is possible that parental expectations regarding appearance may contribute to relationship strain and body dissatisfaction for individuals who strongly identify with traditional Asian values, such as needing parental approval. It is possible that ethnic-racial centrality determines how influential Asian values are on an individual.

The mixed findings on ethnic identity and disordered eating may be attributable to how researchers have defined the construct of acculturation (i.e., extent of assimilation into the dominant culture). Although it is critical to understand the experience of assimilation, assimilation is not exhaustive of the experiences related to ethnic identity, nor of the impact on psychological well-being, such as disordered eating. Previous studies have also not considered how racial identity (i.e., importance of racialized experiences to one's identity) interacts with ethnicity and disordered eating.

### Ethnic-Racial Identity

Ethnic identity is defined as membership in a group with shared cultural traditions, values, and attitudes; racial identity is conceptualized as a membership in a group with shared biological and physical traits, such as skin color (Wilson and Leaper, 2016). Social psychologists have examined racial identity as an important concept that interacts with ethnicity, especially in the United States, coining the term ethnic-racial identity (ERI) (Umana-Taylor et al., 2014). It is defined in three ways: (a) acculturation; (b) ethnic identity (e.g., sense of belonging and positivity to a particular ethnic group); and (c) racial identity (e.g., racial regard, racial ideology, racial centrality). Therefore, the concept ERI encompasses both racial and ethnic experiences, which is an extension of previous research that has focused either on ethnic (Phinney, 1992) or racial identity (Sellers et al., 1998). The present study examined ERI, a synthesis of the constructs of ethnic and racial identity that encapsulates individuals' ethnic background and their racialized experiences (Umana-Taylor et al., 2014). The ethnic-racial identification process includes self-labeling (self and other), ethnic-racial knowledge (e.g., cultural practices), and ethnic racial constancy. This identity process includes specific components such as exploration (i.e., ethnic identity search) and centrality (i.e., group membership is part of your individual identity) (Phinney, 1992; Sellers et al., 1998). With this more expanded construct, a clearer understanding of how ethnic identity impacts disordered eating may be explored.

The purpose of the study was to address this gap in the literature by examining how ERI impacts the relationship between ethnic background and body dissatisfaction. It was hypothesized that ERI would be significantly positively correlated with disordered eating. Furthermore, it was anticipated that ERI centrality (i.e., the importance of ERI) would moderate the relationship between ethnic background and disordered eating (i.e., eating, weight, and shape concerns), such that higher centrality scores will strengthen the positive relationship between ethnic background and disordered eating. It was also predicted that conformity pressure (i.e., the degree to which one feels pressure to conform to one's group) would moderate the relationship between ethnic background and disordered eating (i.e., eating, weight, and shape concerns), such that greater pressures to conform will strengthen the positive relationship between ethnic background and disordered eating.

## Methods

### Participants

Participants were 398 (*M*_age_ = 19.95, *SD* = 3.09) female undergraduates from a large public university in Hawai‘i (Obleada and Bennett, 2021). Participants were recruited through introductory psychology courses and offered partial class credit. Based on the ethnicity with which participants primarily identified, the demographic breakdown was as follows: White (22.9%), Filipino (16.3%), Japanese (16.0%), Chinese (8.4%), Native Hawaiian (6.1%), Hispanic/Latina (5.6%), Korean (3.3 %), Other (4.3%), Other Pacific Islander (2.0%), Black or African American (1.8%), Vietnamese (1.5%), Okinawan (1.3%), and American Indian or Alaska Native (0.5%). Additionally, 12.6% were international students. Notably, in response to a question about ethnic background, 49.1% of participants endorsed being multiethnic.

### Ethnic/Racial Background

Participants were asked to provide demographic information such as age, gender, primary language, and years of education completed. For ethnicity, participants were first asked to indicate all the ethnicities/races that comprised their background. Second, participants were asked to identify their primary ethnicity/race. Individuals who primarily identified as Asian or Caucasian were specifically investigated.

### Disordered Eating

Disordered eating was measured using the Eating Disorder Examination Questionnaire (EDE-Q; Fairburn and Beglin, 2008). The EDE-Q is a 28-question survey that provides a comprehensive assessment of eating disorder psychopathology and related behaviors. The EDE-Q is comprised of four scales: Dietary Restraint (e.g., *Have you tried to exclude from your diet any foods that you like in order to influence your shape or weight?*), Weight Concern (e.g., *Have you had a definite fear that you might gain weight?*), Shape Concern (e.g., *Have you had a definite desire to have a totally flat stomach?)*, and Eating Concern (e.g., *Have you had a definite fear of losing control over eating?*). The four subscales were included to recognize nuances in eating disorder concerns; for example, some individuals are dissatisfied with the contours of their body but do not attend closely to what they weigh, while others focus principally on body weight. A total EDE global score is calculated as the mean of the four scales. EDE-Q subscale items are rated on a seven-point forced-choice format (0–6), with higher scores reflecting greater severity or frequency. In a study of 91 male and female college students, internal consistency was higher for women, with Cronbach's alphas >0.75 at both time 1 and time 2 (Rose et al., 2013). Among women, the 7-day test–retest reliabilities were generally high for all subscales: Restraint (*r* = 0.78), Eating Concern (*r* = 0.83), Shape Concern (*r* = 0.87), and Weight Concern (*r* = 0.90) (Rose et al., 2013).

### Ethnic-Racial Identity

As cited in Wilson and Leaper's (2016) study, Cameron's (2004) social identity scales (centrality, in-group affect, in-group ties) and Egan and Perry's (2001) gender identity scales (felt typicality, felt conformity pressure) were combined to assess ERI. Building on previous measures that assess the ethnic-identity process, this measure captures felt conformity pressure and distinguishes between in-group affect and in-group ties. Items were modeled after the gender identity scales because they appraised an individual's underlying evaluations of in-group membership (Wilson and Leaper, 2016). *Centrality* captures the importance of a particular social identity to one's self-concept. *In-group affect* is how one feels about belonging to a group. *In-group ties* refer to the extent of connectedness to other group members. *Felt typicality* captures how representative one feels oneself to be in reference to other group members. *Felt conformity pressure* is the degree to which one feels pressure to conform to social norms within his or her group. A sample item of the *felt typicality* subscale includes *I have a lot in common with other people within my ethnic/racial group*; items are rated on a five-point Likert scale (1 = *disagree strongly* to 5 = *agree strongly*). In Wilson and Leaper's (2016) study, internal consistency was moderate for each domain: centrality (∝ = 0.79), in-group affect (∝ = 0.83), in-group ties (∝ = 0.79), felt typicality (∝ = 0.75), and felt conformity pressure (∝ = 0.84) based on a sample of 848 undergraduate participants. Reliability for the combined scales was satisfactory (*r* = 0.84; Wilson and Leaper, 2016).

### Procedures and Statistical Analyses

Participants were recruited through the university's SONA (i.e., subject pool management software program) and were given a link to an online survey with an IRB-approved consent form. First, participants were required to review and indicate consent, then fill out a series of computer-based, self-report measures. Upon completion, participants were awarded with research participation credit on SONA. Descriptive analyses and correlations were conducted using the Statistical Package for Social Sciences (SPSS) 26.0. Pearson correlations tested initial associations between all predictor and outcome variables. Using the PROCESS v. 3.3 macro for SPSS, moderation analyses of third variables were conducted (Hayes, 2017).

## Results

### Preliminary Analyses

Caucasian Americans reported higher mean scores on Weight Concern, centrality, and in-group affect compared to Asian Americans; however, when Asian subgroups were examined, it was found that individuals of Vietnamese and Okinawan descent reported the highest mean scores on all outcome measures (see Tables 1, 2, respectively). Pearson correlations revealed that centrality is significantly correlated only with Weight Concern (see Table 3). Consistent with previous literature, Weight Concern was positively associated with centrality *r*_(357)_ = 0.127, *p* < 0.05. Eating Concern and centrality also had a positive correlation, *r*_(357)_ = 0.113, *p* < 0.05. Shape Concern was positively correlated with in-group affect, *r*_(357)_ = 0.174, *p* < 0.01. Weight Concern was also positively associated with in-group affect, *r*_(357)_ = 0.183, *p* < 0.01. The global EDEQ score and in-group affect were positively associated, *r*_(357)_ = 0.147, *p* < 0.01. The datasets presented in this study can be found in an online repository (Obleada and Bennett, 2021).

**Table 1:** Means and standard deviations of outcome measures by ethnic-racial identity.

	**Asian**	**White/Caucasian**
	***M* (*SD*)**	***M* (*SD*)**
EDEQ—weight concern	17.66 (8.63)	18.14 (8.96)
EDEQ—shape concern	3.80 (1.73)	3.75 (1.83)
EDEQ—eating concern	2.18 (1.45)	2.34 (1.46)
ERI—centrality	16.89 (7.88)	34.37 (7.88)
ERI—in-group affect	20.24 (7.00)	35.92 (9.24)

*EDEQ, eating disorder examination questionnaire; ERI, ethnic-racial identity*.

**Table 2:** Means and standard deviations of outcome measures by ethnic-racial identity.

	**EDEQ-WC**	**EDEQ-SC**	**EDEQ-EC**	**Centrality**	**In-group** ** affect**
Chinese (*n* = 33)	15.52 (7.81)	3.38 (1.63)	1.75 (1.03)	12.36 (2.01)	13.79 (2.70)
Filipino (*n* =64)	18.61 (8.62)	4.01 (1.69)	2.35 (1.57)	15.61 (3.08)	18.95 (3.98)
Japanese (*n* = 63)	16.52 (7.99)	3.59 (1.62)	1.97 (1.21)	16.98 (3.03)	20.50 (5.20)
Okinawan (*n* = 5)	22.80 (12.62)	4.69 (2.24)	3.68 (2.26)	23.10 (7.67)	29.75 (5.80)
Korean (*n* = 13)	19.69 (9.71)	4.07 (2.13)	2.57 (1.75)	25.23 (5.69)	29.85 (5.68)
Vietnamese (*n* = 6)	22.33 (11.33)	4.85 (2.09)	2.90 (1.95)	31.17 (10.82)	38.04 (9.90)
White (*n* = 90)	18.14 (8.96)	3.75 (1.83)	2.34 (1.46)	34.37 (7.88)	35.92 (9.24)

*EDEQ-WC, eating disorder examination questionnaire weight concern subscale; EDEQ-SC, eating disorder examination questionnaire shape concern subscale; EDEQ-EC, eating disorder examination questionnaire eating concern subscale*.

**Table 3:** Pearson's correlations for all outcome measures.

	**EDEQ-WC**	**EDEQ-SC**	**EDEQ-EC**	**Centrality**	**In-group** ** affect**
EDEQ-WC	–	–	–	–	–
EDEQ-SC	0.94^***^	–	–	–	–
EDEQ-EC	0.73^***^	0.73^***^	–	–	–
Centrality	0.08	0.04	0.15^*^	–	–
In-group affect	0.13^*^	0.10	0.15^*^	0.80^***^	–

*EDEQ-WC, eating disorder examination questionnaire weight concern subscale; EDEQ-SC, eating disorder examination questionnaire shape concern subscale; EDEQ-EC, eating disorder examination questionnaire eating concern subscale*.

* *p < 0.05*,

** *p < 0.01*,

*** *p < 0.001 (Johnstone, 1987)*.

### Moderation Analyses

Primary ethnic identification and ERI were used to predict disordered eating. Each predictor was mean centered, and the interaction between primary ethnic identification and ethnic racial identity was analyzed using Hayes' PROCESS plug-in for SPSS (Hayes, 2017).

### Centrality as a Moderator Between Ethnicity and Eating Concern

The main effects of primary ethnic identification and centrality were significant predictors of EDEQ Eating Concern *F*_(5, 347)_ = 2.42, *p* = 0.036, *R*^2^ = 0.03. Both identifying as Asian and identifying as Caucasian were not significantly related to Eating Concern (see Table 4). Eating Concern was not significantly related to centrality of race/ethnicity. Eating Concern was not significantly predicted by the interaction between ethnicity and centrality with identifying as Caucasian. However, Eating Concern was significantly predicted by the interaction between ethnicity and centrality with those identifying as Asian, *b* = 0.05, *t*_(347)_ = 2.37, *p* = 0.018.

**Table 4:** Linear models of predictors on outcome variables.

**Path**	***b***	**df**	***t***	***p***
**Centrality and eating concern**				
Centrality (M)	0.01	347	0.55	0.579
Identifying as Asian (X_1_)	−0.86	347	−1.54	0.124
Identifying as Caucasian (X_2_)	−0.28	347	−0.34	0.734
Centrality × identifying as Asian (interaction_1_)	0.05	347	2.37	0.018^*^
Centrality × identifying as Caucasian (interaction_2_)	0.01	347	0.47	0.642
**Centrality and weight concern**				
Centrality (M)	0.13	345	1.66	0.098
Identifying as Asian (X_1_)	−2.90	345	−0.86	0.388
Identifying as Caucasian (X_2_)	6.57	345	1.36	0.176
Centrality × identifying as Asian (interaction_1_)	0.23	345	1.68	0.095
Centrality × identifying as Caucasian (interaction_2_)	−0.22	345	−1.55	0.123
**In-group affect and weight concern**				
In-group affect (M)	0.15	345	2.33	0.021^*^
Identifying as Asian (X_1_)	−1.37	345	−0.41	0.683
Identifying as Caucasian (X_2_)	5.19	345	1.15	0.252
Centrality × identifying as Asian (interaction_1_)	−0.16	345	1.41	0.158
Centrality × identifying as Caucasian (interaction_2_)	−0.15	345	−1.26	0.210
**In-group affect and shape concern**				
In-group affect (M)	0.03	347	2.31	0.022^*^
Identifying as Asian (X_1_)	−0.10	347	−0.15	0.878
Identifying as Caucasian (X_2_)	0.86	347	0.94	0.346
Centrality × identifying as Asian (interaction_1_)	0.03	347	1.28	0.200
Centrality × identifying as Caucasian (interaction_2_)	−0.03	347	−1.05	0.292

*Centrality and eating concerns model R^2^ = 0.03; centrality and weight concerns model R^2^ = 0.04; in-group affect and weight concerns model R^2^ = 0.05; in-group affect and shape concerns model R^2^ = 0.04*.

* *p < 0.05*,

** *p < 0.01*,

*** *p < 0.001 (Johnstone, 1987)*.

### Centrality as a Moderator Between Ethnicity and Weight Concern

The main effects of primary ethnic identification and centrality were significant predictors of EDEQ Weight Concern, *F*_(5, 345)_ = 2.79, *p* = 0.018, *R*^2^ = 0.04. Identifying as Asian was not significantly related to Weight Concern nor was identifying as Caucasian. Weight Concern was not significantly related to centrality and was not predicted by the interaction between ethnicity and centrality if identifying as Asian or Caucasian (see Table 4).

### In-group Affect as a Moderator Between Ethnicity and Weight Concern

The main effects of primary ethnic identification and in-group affect were significant predictors of Weight Concern, *F*_(5, 345)_ = 3.68, *p* = 0.003, *R*^2^ = 0.05. Neither identifying as Asian nor identifying as Caucasian was significantly related to Weight Concern. Weight Concern was significantly related to in-group affect, *b* = 0.15, *t*_(345)_ = 2.33, *p* = 0.021. However, the moderation was not significant as Weight Concern was not significantly predicted by the interaction between ethnicity and in-group affect, when identifying as Asian or when identifying as Caucasian (see Table 4).

### In-group Affect as a Moderator Between Ethnicity and Shape Concern

The main effects of primary ethnic identification and in-group affect were significant predictors of EDEQ Shape Concern, *F*_(5, 347)_ = 3.22, *p* = 0.008, *R*^2^ = 0.04. Identifying as Asian was not significantly related to Shape Concern nor was identifying as Caucasian. Shape Concern was significantly related to in-group affect, *b* = 0.03, *t*_(347)_ = 2.31, *p* = 0.022. However, the moderation was not significant as Shape Concern was not significantly predicted by the interaction between ethnicity and in-group affect, if identifying as Asian nor when identifying as Caucasian (see Table 4).

## Discussion

The current study examined the interrelationship between primary ethnic identification, ERI, and disordered eating. Consistent with the first hypothesis, ERI was significantly, positively correlated with disordered eating (i.e., eating, weight, and shape concerns); specifically, as centrality (i.e., importance of a particular social identity to one's self-concept) increased, eating, weight, and shape concerns increased. The second hypothesis was partially supported, such that centrality moderated the effect of primary ethnic identification on eating concerns when identifying as Asian. Interestingly, this finding suggests that for Asian American individuals, the degree to which eating concerns are distressing may be moderated by how important ethnicity is to one's identity. This effect supports previous findings that have identified family eating concerns as a possible risk factor, particularly for Asian Americans (Smart and Tsong, 2014). An examination of existing literature on cultural beliefs in disordered eating among Asian American women has identified specific values related to family recognition through achievement and emotional self-control as significant predictors of disordered eating (Tsong and Smart, 2015). Additionally, Han (2020) found that tensions from pursuing family recognition and intergenerational conflict influenced binge-eating and restriction only when certain psychological needs (e.g., autonomy, competence, connectedness) were not met, thus implying that this phenomenon may be more nuanced.

In addition to possible family influences, centrality of Asian identity related to distress about eating concerns in Asian American women may be due to appearance pressures and body ideals being central to Asian identity. For example, past research found that Asian women compared to women in countries across Europe, the Mediterranean, and South America held stricter standards about achieving the values of thinness compared to other ethnicities (Wardle et al., 2006). It is possible that individuals who find their Asian race more central to their overall identity could be more likely to subscribe to these values about achieving thinness. Previous findings have also shown that some Asian values, particularly conformity to group norms and family recognition through achievement, are associated with disordered eating among Asian American women (Tsong and Smart, 2015; Han, 2020). According to symbolic interaction theory, individuals develop their self-concept based on reflected appraisals (i.e., beliefs about how they are perceived by others), such that their identity is partly construed by perceptions or evaluations that others have of them (Alvarez and Helms, 2001; Aksan et al., 2009). This theory may help to explain why Asian cultures that value conforming to group norms find it especially important to adhere to rules of conduct—because it is integrated into their identity. For Asian culture's collectivistic values of family recognition through achievement and conforming to group norms, symbolic interaction helps to explain the importance that cultural values may play in Asian identity. Therefore, if an Asian American individual highly appraises the values of their family's culture of origin, then it may be important for their self-concept to adhere to these ideals including standards about beauty.

Additionally, though in-group affect was not identified as a moderator, the results suggest that greater in-group affect predicted both greater Shape Concern and greater Weight Concern regardless of one's primarily identified ethnicity. Of particular note is how results demonstrated that culture is important to the Caucasian Americans sampled in this study. It is not clear why in-group affect would be important to these individuals; however, specific cultural aspects related to family country of origin (e.g., Italian, Jewish ancestry) were not assessed and merit further investigation into aspects of eating related to the connectedness of culture. Since almost 50% of the sample identified as being multiethnic, some Caucasian Americans may also have a multiethnic background that possibly contributed to their culture's influence on eating concerns.

## Limitations and Future Directions

The first limitation of this study is that it collapsed all Asians ethnicities into one group of Asian Americans for analyses. The demographic breakdown of our participants suggests that the individuals who identified as Asian primarily come from East Asian backgrounds. Therefore, the outcomes are likely more reflective of the experience of East Asian Americans, rather than all Asian Americans. Future research should examine differences in the relationships between ethnic centrality and disordered eating variables across different countries of origin across Asia and Europe. Additionally, research has shown that there are particular nuances of disordered eating concerns within all Asian ethnicities (Lee et al., 1989, 2010; Edman and Yates, 2005; Jackson et al., 2006; Nakai et al., 2014a). Future research should examine underlying mechanisms that account for the influences of ERI on eating concerns and examine when ethnic populations are more affected by this moderation relationship. Another limitation is that the paucity of research within this population could contribute to measurement inaccuracies. While the measures used in the present study had adequate internal consistency and are considered to be valid and reliable more broadly, their psychometric properties have not been examined in a sample with similar ethnic and racial diversity. For example, in the present study, we used a standard measurement tool for assessing eating behaviors, the EDE-Q; however, past research suggests that norms may differ across racial groups (Nakai et al., 2014b). Future research should examine whether this measure is the best fit for assessing eating pathology in diverse samples. Additionally, it may be important for clinicians and researchers to consider how the importance of ethnic identity and its manifestation within disordered eating may affect particular Asian American subgroups, such that understanding these differences may be especially important in informing culturally sensitive treatment and prevention approaches for eating pathology. Given that the results from this study include a multiethnic sample, subsequent research examining the differences between multiethnic and single ethnic identities within the context of eating pathology is also needed. Finally, further research should explore how other aspects of culture may contribute to the value or importance of eating concerns among Caucasian individuals.

## Conclusions

This study is one of the first to examine centrality within the context of disordered eating concerns. Of the ERI subscales, only centrality had a moderation effect on eating concerns; however, it may also be important to explore how centrality may impact other eating disorder constructs that have been found to be influenced by race/ethnicity, such as body surveillance or drive for thinness. It may also be desirable for clinicians and researchers to consider how the importance of ethnic identity and its manifestation within disordered eating may affect particular ethnic groups. In this study, the effect was more pronounced for Asian Americans compared to Caucasian Americans. Lastly, while the results provide initial evidence that ethnic centrality is potentially an important factor in explaining some of the notable differences in disordered eating pathology between American women who identify as Asian vs. Caucasian, the findings of the present study truly underscore the need for further research across racial and ethnic groups in order to better understand the nuanced differences in experiences of cultural ideals and disordered eating.

## Data Availability Statement

The names of the repository/repositories and accession number(s) can be found below: https://doi.org/10.6084/m9.figshare.12800066.v1.

## Ethics Statement

The studies involving human participants were reviewed and approved by University of Hawaii at Manoa Human Studies Program. The patients/participants provided their written informed consent to participate in this study.

## Author Contributions

KO contributed to conception and design of the study and organized the database. KO and BB performed the statistical analysis. KO wrote the first draft of the manuscript. Both authors contributed to manuscript revision, read, and approved the submitted version.

## Conflict of Interest

The authors declare that the research was conducted in the absence of any commercial or financial relationships that could be construed as a potential conflict of interest.
